# AGEISM AND PERSONALITY AS UNIQUE PREDICTORS OF OLDER ADULT WELLBEING

**DOI:** 10.1093/geroni/igae098.4116

**Published:** 2024-12-31

**Authors:** Claire Wininger, Megan Wolk, Jennifer Beatty, Judy Kwak, Patrick Hill

**Affiliations:** Washington University in St. Louis, St. Louis, Missouri, United States; Washington University in St. Louis, St. Louis, Missouri, United States; Washington University in St. Louis, St. Louis, Missouri, United States; Washington University in St. Louis, St. Louis, Missouri, United States; Washington University in St. Louis, St. Louis, Missouri, United States

## Abstract

Ageism is a primary risk factor for poorer psychological wellbeing in older adulthood. However, the pernicious effects of ageism prove complicated to alleviate given they move beyond whether one experiences discrimination due to age, as older adults often internalize the negative stereotypes associated with these discriminatory acts. The current study evaluated whether certain personality traits may exacerbate or mitigate the wellbeing concerns associated with ageism. Participants (n = 250, ages: 60-80) completed measures of ageist discrimination, internalized ageism, the Big Five personality traits, and psychological wellbeing. Results indicated that older adults who reported greater ageist discrimination and internalized ageism reported worse psychological wellbeing, as did adults who scored lower on extraversion but higher on neuroticism. Multiple regression tests of moderation though found that the Big Five personality traits failed to moderate ageism-wellbeing associations. Put differently, personality traits and ageism were independent predictors of wellbeing. Moreover, the current study found that experiences of ageist discrimination and internalized ageism were positively correlated, and both held unique negative associations with psychological wellbeing. These findings clearly demonstrate that both forms of ageism hold independent pernicious effects for older adults largely regardless of their personality.

